# The Influence of the COVID-19 Pandemic on Patients Admitted with Pericardial Effusion

**DOI:** 10.3390/diagnostics16030464

**Published:** 2026-02-02

**Authors:** Amir Shabtay, Iftach Sagy, Elizaveta Rabaev, Hezzy Shmueli, Leonid Barski

**Affiliations:** 1 Cardiology Division, Soroka University Medical Center, P.O. Box 151, Be’er Sheva 84101, Israel; hezzysh@clalit.org.il; 2 Faculty of Health Sciences, Ben-Gurion University of the Negev, 1 Ben Gurion Boulevard, Be’er Sheva, Israel; iftachsagy@gmail.com (I.S.); lizarabaev@gmail.com (E.R.); lbarski@bgu.ac.il (L.B.); 3 Rheumatology Unit, Soroka University Medical Center, P.O. Box 151, Be’er Sheva 84101, Israel; 4 Clinical Research Center, Soroka University Medical Center, P.O. Box 151, Be’er Sheva 84101, Israel; 5 Internal Medicine Division, Soroka University Medical Center, P.O. Box 151, Be’er Sheva 84101, Israel

**Keywords:** COVID-19, pericardial effusion, pericardiocentesis, point-of-care ultrasound

## Abstract

**Background**: SARS-CoV-2 infection, its late complications, and SARS-CoV-2 vaccines are known to cause pericardial effusion. We sought to investigate the influence of the COVID-19 outbreak on trends in pericardiocentesis. **Methods**: We performed a retrospective population study including all >18 years patients undergoing pericardiocentesis in a single tertiary hospital between January 2018 and April 2022. The effusion characteristics and patient outcomes were compared between patients admitted before and after the COVID-19 outbreak. **Results**: 92 patients underwent pericardiocentesis cases during the COVID-19 period compared to 65 patients during the pre-COVID-19 period (*χ*^2^ = 3.07, *p* = 0.0796). Only 15% of the post-COVID-19 outbreak cases were related to COVID-19 infection or vaccine. In-hospital mortality was numerically higher during the post-COVID-19 group (7.7% vs. 14.4%), but this difference did not reach statistical significance (*p* = 0.22). The 90-day mortality was also similar between groups. **Conclusions**: A numerical, yet statistically insignificant increase in pericardiocentesis was observed following the COVID-19 outbreak. We assume this observation cannot be attributed solely to the virus and vaccines per se. Neglect of other chronic diseases, social distancing, and widespread availability of point-of-care ultrasound may have contributed to this observation.

## 1. Introduction

Pericardial effusion may be a common finding in clinical practice [1]. Symptoms might include cardiac tamponade, chest pain, dyspnea, and pedal edema. Still, it can also be an unexpected finding in imaging studies.

Some established causes for pericardial effusion include infectious pericarditis, end-stage kidney disease, neoplasms, or connective tissue diseases. Many cases, however, remain idiopathic [2]. Pericardiocentesis is an important step in managing patients with pericardial effusion. It promotes both diagnosis and symptom-relief.

SARS-CoV-2 was reported as a causative agent for pericardial effusion in a previously published case series [3]. Pericardial effusion and pericarditis were also late complications among COVID-19-recovered patients [4]. Some SARS-CoV-2 vaccines were also reported to cause pericarditis and pericardial effusion, particularly among children and adolescents [5].

The COVID-19 pandemic could, however, lead to change in pericardial effusion cases by other means: social distancing and the use of face masks could have caused a reduction in the incidence of other respiratory infections that cause pericarditis. Patients with chronic kidney disease or cancer might have refrained from medical care, therefore risking themselves in disease progression and pericardial involvement [6].

The present study aims to analyze temporal trends in the incidence and etiology of pericardiocentesis. We also seek to evaluate whether the COVID-19 pandemic is associated with a change in the number of pericardiocentesis procedures in the observed etiology of pericardial effusion and a difference in the pericardiocentesis-related mortality.

## 2. Materials and Methods

### 2.1. Study Design

We performed a retrospective chart review of patients admitted to Soroka University Medical Center (SUMC) between January 2018 and April 2022. SUMC is the only tertiary medical center in Southern Israel. It serves over 700,000 people living in this region and is also a referral center for medical centers from nearby areas.

SUMC Institutional Review Board approved this study.

Patients were stratified into two groups: (1) Patients admitted between January 2018 and February 2020 (Pre COVID-19 group), and (2) Patients admitted between March 2020 and April 2022 (Post COVID-19 Outbreak group).

We compared the number of pericardiocentesis procedures performed alongside demographic parameters, pre-existing medical conditions, patient survival, pericardial fluid histopathologic findings, and the etiology for pericardial fluid across groups.

### 2.2. Patient Population

All patients aged 18 years or older, admitted to SUMC who had undergone pericardiocentesis were included. We considered only the first event for the statistical analysis. Patients undergoing pericardiocentesis due to traumatic pericardial effusion and repeated pericardiocentesis were excluded.

### 2.3. Endpoints and Terminology

**Figure 1:**
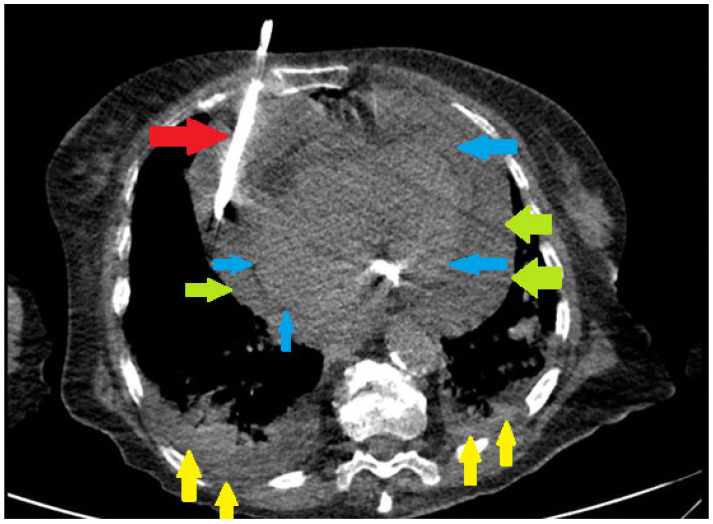
Pericardial drain insertion in a patient with COVID-19 infection, large pericardial effusion, and bilateral pneumonia. Red arrow—pericardial drain. Green arrows—parietal pericardium. Blue arrows—visceral pericardium. Yellow arrows—pulmonary infiltrate.

Pericardiocentesis was defined as needle aspiration of pericardial fluid or any surgical procedure involving drainage of pericardial fluid, including surgical pericardiotomy and pericardial window (Figure 1). Patient records with ICD code of pericardiocentesis, pericardiotomy or pericardiectomy were thoroughly reviewed by the investigators, to ensure that drainage of pericardial fluid was performed.

Patient records with ICD code of pericardiocentesis or cardiac tamponade were viewed by the investigators. The presence of cardiac tamponade was determined by documentation of clinical (Beck’s Triad) or sonographic signs as were documented by a thorough echocardiography test (right atrial/ventricular systolic collapse, mitral flow variation) [7].

**Figure 2:**
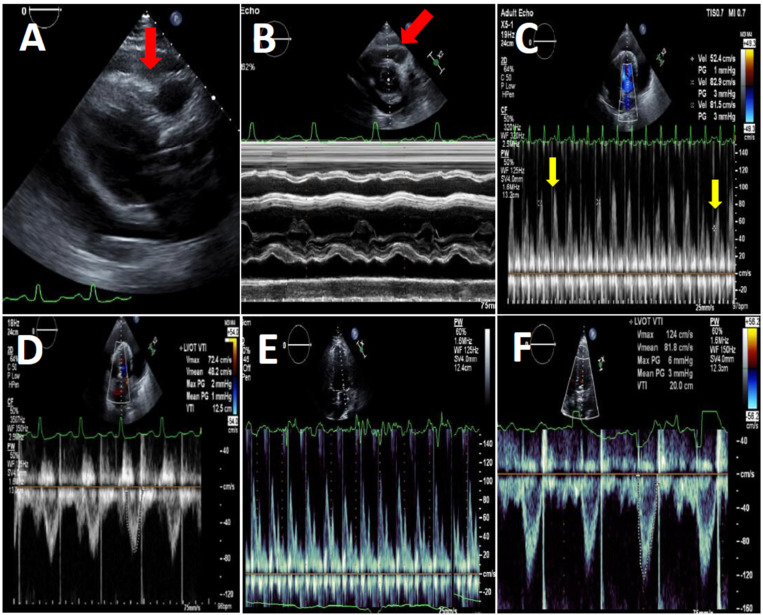
Transthoracic echocardiography of a COVID-19 patient before (panels (**A**–**D**)) and after pericardiocentesis (panels (**E**,**F**)). (**A**)—Parasternal long axis view, diastolic RV free wall collapse (red arrow) due to high pressure in the pericardial space, due to a large circumferential pericardial effusion. (**B**)—Short axis view M-Mode at the level of the aortic valve showing diastolic collapse of the RV free wall (red arrow). (**C**)—Apical 4-chamber view PW Doppler of the mitral flow showing respiratory variations between beats (yellow arrows). (**D**)—Apical 4-chamber view PW Doppler of the left ventricular outflow tract systolic flow showing low VTI, indicating low stroke volume. (**E**)—Apical 4-chamber view PW Doppler of the mitral flow showing normalization of mitral flow pattern. (**F**)—Apical 4-chamber view PW Doppler of the LVOT systolic flow showing normal VTI, indicating normal stroke volume.

Left ventricular function and effusion size were determined by echocardiography (Figure 2). All studies were performed by experienced sonographers and were interpreted by senior cardiologists specialized in echocardiography [8,9]. All studies were performed with the VIVID E3 95 Ultrasound device manufactured by General Electric and were held following the standard protocol of the American Society of Echocardiography guidelines.

**Figure 3:**
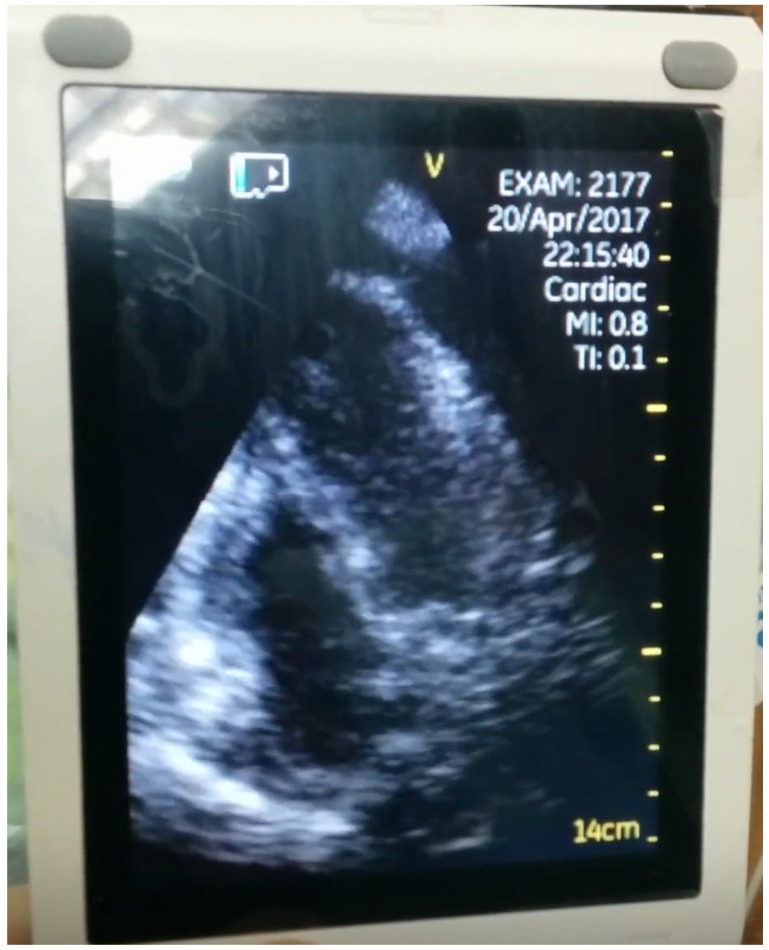
Point-of-care ultrasound of a patient with clinical signs of tamponade. The picture is a screenshot from a mobile phone, as at this point of care ultrasound devices in our institution do not produce DICOM images but rather allow real-time imaging only.

We also documented bedside point-of-care-ultrasaound (POCUS), if performed, in the initial evaluation of the patients before conducting the echocardiography. POCUS was performed using a General Electric Venue Go or a handheld V-Scan ultrasound device (Figure 3).

SARS-CoV-2 positive status was defined as a positive PCR or antigen assay performed by a medical professional and documented in the electronic medical record.

In-hospital mortality was defined as death before discharge or within 30 days of admission, even if it occurred outside of the hospital. 90-day mortality was recorded using the same method.

COVID-19-related pericardial fluid was defined as fluid occurring within 60 days of SARS-CoV-2 infection or vaccine without any other identifiable etiology.

According to the pathological report, a malignant pericardial effusion was determined if the pericardial fluid contained neoplastic cells or if a pericardial biopsy demonstrated neoplastic cells.

Infectious pericarditis was defined as typical symptoms (pleuritic chest pain, fever) with supporting laboratory findings (elevated erythrocyte sedimentation rate, C-reactive protein, positive culture, positive viral PCR or immunoassays).

Peri-procedural pericardial effusion was defined as bloody or inflammatory pericardial fluid occurring within 30 days of a surgical or percutaneous procedure involving the heart (e.g., valve replacement surgery).

### 2.4. Statistical Analysis

Data are expressed as mean ± standard deviation (SD), median ± interquartile range (IQR), or number and percentage. We compared patient characteristics between pre-COVID-19 vs. post-COVID-19 admissions using t-test, chi-square, and non-parametric tests. We plotted the 90-day mortality using the Kaplan–Meier curve stratified by pre-COVID-19 vs. post-COVID-19, calculating the *p*-value of log-rank. Statistical analysis was performed using SPSS version 25.0.

## 3. Results

We identified 162 patients who underwent pericardiocentesis during the study period. The general characteristics of patients stratified before and after the COVID-19 outbreak are presented in Table 1. No significant difference was observed between groups in baseline socio-demographic parameters and comorbidities. However, a 49.2% rise in pericardiocentesis cases was observed in the post-COVID-19 group (*n* = 92 vs. 65 in the pre-COVID-19 group). However, this difference did not reach statistical significance (*χ*^2^ = 3.07, *p* = 0.0796). Common chief complaints prior to pericardiocentesis included dyspnea and chest pain.

**Figure 4:**
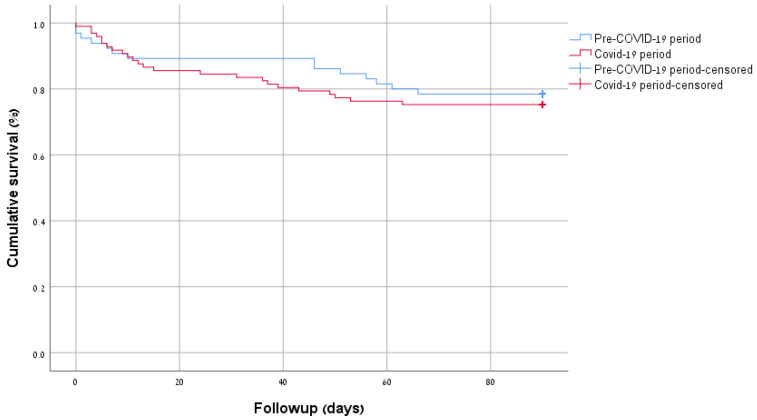
Kaplan–Meier of cumulative survival by period (*p* = 0.60).

In-hospital mortality was numerically higher in the post-COVID-19 group (7.7% vs. 14.4%, *p* = 0.22), but this difference was not statistically significant. Moreover, 90-day mortality (21.5% vs. 24.7%, *p* = 0.70) and Kaplan–Meier survival curves (*p* = 0.60) (Figure 4) did not demonstrate a significant difference between groups. Among 15 COVID-19-related pericardiocentesis cases, 2 died in-hospital, a proportion comparable to the in-hospital mortality rate of the general study population.

Large pericardial effusion and cardiac tamponade were highly prevalent in both groups, and prevalence did not vary significantly (Table 2). COVID-19 infection or vaccine accounted for 15.5% of pericardiocentesis cases in the post-COVID-19 group. Consequently, the overall relative prevalence of other effusion etiologies varied significantly between groups (*p* = 0.04). On the other hand, histopathology findings did not differ significantly between groups (*p* = 0.22).

Point-of-care ultrasound (POCUS) was performed in 39.7% of pre-COVID-19 patients and 49.5% of post-COVID-19 patients (*p* = 0.25). The prevalence of sonographic signs of cardiac tamponade did not vary significantly between groups (Table 2). Time from POCUS to diagnosis and from POCUS to pericardiocentesis did not vary between groups as well.

## 4. Discussion

Our study aimed to assess the influence of the COVID-19 outbreak on patients admitted with pericardial effusion and who underwent pericardiocentesis. An almost 50% rise in pericardiocentesis cases, attributable only in part to COVID-19 infection or vaccine, was demonstrated. This rise, however, was not statistically significant. Notwithstanding, other covariates, including socio-demographic parameters and comorbidities, did not vary significantly between groups.

SUMC is the only tertiary medical center in Southern Israel, and therefore the single healthcare provider to a population of almost 400 thousand adults in the region. Due to the urgent nature of the procedure, it would be unlikely that a change in prevalence is attributable to patients from other regions in Israel who chose to be admitted to SUMC, or that patients from this region will seek medical attention outside the region in the case of clinically significant pericardial effusion.

COVID-19 may have contributed to a change in pericardiocentesis cases by various indirect mechanisms. Therefore, we assume that pericardiocentesis cases related to COVID-19 represent only part of a broader phenomenon. One such mechanism could be patients refraining from treatment or medical advice following government-ordered lockdowns, fear of contracting COVID-19 while in the hospital, or some medical services (such as CT or echocardiography deemed non-urgent) being restricted during the pandemic [10,11]. Such a phenomenon was demonstrated in the USA, including some medical emergencies [12]. Patients refraining from medical care might risk themselves with cancer or renal failure progression unnoticed by medical professionals [13,14,15], eventually postponing their diagnosis and consequently presenting with pericardial effusion. Conversely, some patients might die outside the hospital due to medical urgency, not receiving appropriate attention. The low use of autopsy in Israel makes it hard for investigators to know whether patients found dead in their homes had a clinically significant pericardial effusion.

The lack of long and short-term survival gaps between groups suggests the hypothesis of refraining from medical care during the pandemic is less plausible.

Social distancing and the widespread use of face masks could have prevented the spread of respiratory viruses causing viral pericarditis [16,17], and might account for the lack of statistically significant rise in pericardiocentesis cases despite the addition of COVID-19 related cases. However, the proportion of infectious (non-COVID-19) pericarditis did not differ between groups, making this hypothesis also unlikely.

It is known that SARS-CoV-2 can cause or increase immune dysregulation and autoimmunity [18,19], which can present as serositis, including pericardial effusion. However, the prevalence of connective tissue diseases and HIV among patients in our study was low, and most of those patients did not contract COVID-19 during the study period. Whether pericardial effusion was a long-term complication of recent COVID-19 infection warrants further investigation. Our study is unable, by nature, to establish a causal relationship between those conditions.

Point-of-care ultrasound (POCUS) devices were widely deployed in our hospital during the first half of 2020, with many physicians being trained to use them. Detection of large pericardial effusion using POCUS is relatively easy [20]. It could be hypothesized that POCUS led to overdiagnoses of pericardial effusion, leading to unnecessary pericardiocentesis. However, an overwhelming majority of patients in our study had either large pericardial effusion or cardiac tamponade, which are relatively easier to diagnose in the absence of POCUS as compared to small pericardial effusion (e.g., by clinical signs or using chest X-ray).

While POCUS aided diagnosis in a numerically higher proportion of patients in the post-COVID-19 group (Table 2), this difference did not reach statistical significance (39.7% vs. 49.5%, *p* = 0.25), nor did it lead to a more frequent diagnosis of cardiac tamponade (9.5% vs. 9.3%, *p* = 1).

Despite a previous small study suggesting that POCUS could shorten the admission to pericardiocentesis interval [21], it did not affect admission to pericardiocentesis or symptoms to pericardiocentesis intervals in our study (Table 2). This could represent, in part, the urgent nature of pericardiocentesis in many of the patients in our study (due to clinical signs of tamponade), prompting the use of echocardiography or CT to aid in drainage.

Considering the above and the fact that large effusions or tamponades are absolute indications for pericardiocentesis, it would be unlikely that POCUS caused overdiagnosis of pericardial effusion or led to excess procedures. Moreover, the absence of a survival gap between groups further strengthens our opinion that pericardiocentesis was performed promptly and following accepted indications in both groups.

Regarding survival, the total number of in-hospital deaths in the post-COVID-19 group was numerically doubled compared to the pre-COVID-19 group. Still, the final numbers in both groups are low, and the difference is not statistically significant. The lack of difference in 90-day mortality and Kaplan–Meier survival curves between groups suggests that survival did not differ. However, it is possible that our study was underpowered to detect such a difference in in-hospital mortality.

Our study has several limitations that provide opportunities for future research. First, this is a single-center study representing mainly two population groups (Jewish and Bedouin-Arab people), consisting of most of the population in Southern Israel. Hence, we cannot assume causality, and the generalizability of our results is limited. However, by utilizing data from a single tertiary center—the sole provider for the region’s population—we ensured a high degree of data consistency and procedural uniformity that larger, multi-center registries and studies often lack.

Moreover, the absolute number of in-hospital deaths is low. Pericardiocentesis is a relatively uncommon procedure. Thus, despite an apparent large numerical increase in in-hospital mortality during COVID-19, a larger cohort may be more powered to detect statistically significant changes in mortality. The observed meaningful increase in pericardiocentesis cases is hypothesis-generating and should be further investigated in multi-center studies.

Some COVID-19-related complications also appear as a late sequel of the infection [22,23]. Our study’s data gathering ended in April 2022, most COVID-19 cases in Israel occurred between collection completed in April 2022, and most COVID-19 cases in Israel occurred between late 2021 and early 2022. It is possible that some long-term ramifications of the COVID-19 infection or vaccine did not appear within this time frame. It is also possible that long-term consequences of loss to medical follow-up during lockdown periods are yet to occur due to the slow progression of many cancers, for example. Large-scale multicenter studies might detect a statistically significant change in the number of pericardiocentesis cases or their etiology.

## 5. Conclusions

Our study aimed to evaluate trends in pericardiocentesis amidst the COVID-19 pandemic outbreak in Israel. We observed a numerical, although not statistically significant, rise in pericardiocentesis cases following the pandemic outbreak. This observation is probably not attributed solely to SARS-CoV-2 infection or vaccination. Neglect of other chronic diseases, social distancing, and increased availability of point-of-care ultrasound may have contributed. We hope that future larger-scale studies will confirm our results.

## Figures and Tables

**Table 1:** Cohort characteristics by the period of pericardiocentesis.

Variable	Pre-COVID-19 Period (*n* = 65)	COVID-19 Period (*n* = 97)	*p* Value
Age (mean ± SD)	62.3 (2.0)	65.4 (1.6)	0.23
Females (*n*,%)	35 (53.8)	40 (41.2)	0.14
Jews (*n*,%)	52 (80.0)	81 (83.5)	0.67
Socio-economic status (*n*,%)			
Low	27 (45.8)	29 (34.9)	0.220
Medium	23 (39.0)	32 (38.6)	
High	9 (15.3)	22 (26.5)	
Smokers (*n*,%)	22 (34.9)	38 (41.3)	0.50
Heart failure(*n*,%)	10 (15.4)	23 (23.7)	0.23
Ischemic heart disease (*n*,%)	10 (15.4)	19 (19.6)	0.53
Diabetes (*n*,%)	31 (47.7)	37 (38.1)	0.25
Chronic kidney disease (*n*,%)	20 (30.8)	28 (28.9)	0.86
Connective tissue disease (*n*,%)	6 (9.2)	5 (5.2)	0.35
Malignancy (*n*,%)	22 (33.8)	32 (33.0)	1.00
Dementia (*n*,%)	2 (3.1)	2 (2.1)	1.00
Charlson comorbidity index (median, interquartile range)	6.0 (3.0–9.0)	6.0 (4.0–9.0)	0.71
Symptoms to pericardiocentesis, days (median, interquartile range)	1.0 (1.0–2.0)	1.0 (1.0–2.0)	0.88
Chief complaint (*n*,%)			
Chest pain	8 (22.9)	9 (15.0)	0.11
Dyspnea	9 (25.7)	26 (43.3)	
Other	6 (17.1)	12 (20.0)	
Could not determine	12 (34.3)	13 (21.7)	
Length of hospitalization (median, interquartile range)	7.0 (4.0–10.5)	6.0 (4.0–9.7)	0.55
ICU admission (*n*,%)	51 (78.5)	69 (71.1)	0.36
In-hospital mortality (*n*,%)	5 (7.7)	14 (14.4)	0.22
90-day mortality (*n*,%)	14 (21.5)	24 (24.7)	0.70

**Table 2:** Pericardial effusion fluid and echocardiographic characteristics.

Variable	Pre-COVID-19 Period (n = 65)	COVID-19 Period (n = 97)	*p* Value
Large effusion size (*n*,%)	52 (81.3)	73 (75.3)	0.44
Tamponade *n* (%)	35 (54.7)	63 (64.9)	0.24
Reduced left ventricular systolic function *n* (%)	16 (25.0)	19 (19.6)	0.44
Reduced right ventricular systolic function *n* (%)	12 (25.0)	12 (16.0)	0.24
Fluid pathology *n* (%)			
Inflammatory	27 (42.2)	40 (41.2)	0.22
Bloody	3 (4.7)	13 (13.4)	
Neoplastic	10 (15.6)	16 (16.5)	
Pyogenic	0 (0.0)	2 (2.1)	
POCUS done n (%)	25 (39.7)	48 (49.5)	0.25
Tamponade on POCUS *n* (%)	6 (9.5)	9 (9.3)	1.00
POCUS to diagnosis, days (median, interquartile range)	1.0 (1.0–1.0)	1.0 (1.0–1.0)	0.88
POCUS to pericardiocentesis, days (median, interquartile range)	1.0 (1.0–2.0)	1.0 (1.0–1.0)	0.10
Final etiology			
Malignant	16 (24.6)	17 (17.5)	0.04
Peri-procedural	11 (16.9)	14 (14.4)	
Infectious	12 (18.5)	17 (17.5)	
COVID-19 related	0 (0)	15 (15.5)	
Idiopathic	18 (27.7)	24 (24.7)	
Other	8 (12.3)	10 (10.3)	

## Data Availability

The original contributions presented in this study are included in the article. Further inquiries can be directed to the corresponding author.

## Abbreviations

The following abbreviations are used in this manuscript:

COVID-19	Coronavirus disease 19
CT	Computed tomography
ICD	International classification of disease
IQR	Interquartile range
PCR	Polymerase chain reaction
POCUS	Point-of-care ultrasound
SARS-CoV-2	Severe acute respiratory syndrome Coronavirus 2
SD	Standard deviation
SUMC	Soroka University Medical Center
USA	United States of America
